# Association between diabetes, obesity, and quality of life in preoperative patients with degenerative cervical myelopathy: A cross‐sectional study

**DOI:** 10.1002/hsr2.70005

**Published:** 2024-08-20

**Authors:** Yasuaki Mizoguchi, Kiyokazu Akasaka, Kenta Suzuki, Fumihiko Kimura, Toby Hall, Satoshi Ogihara

**Affiliations:** ^1^ Saitama Medical University Graduate School of Medicine Saitama Japan; ^2^ Department of Rehabilitation Kimura Orthopaedic Clinic Saitama Japan; ^3^ School of Physical Therapy, Faculty of Health and Medical Care Saitama Medical University Saitama Japan; ^4^ Curtin School of Allied Health Curtin University Perth Australia; ^5^ Tokyo Spine Hospital Tokyo Japan

**Keywords:** cervical cord, diabetes mellitus, obesity, patient reported outcome measures, preoperative care, quality of life

## Abstract

**Background and Aims:**

Degenerative cervical myelopathy (DCM) is a debilitating condition characterized by compression of the cervical spinal cord, leading to neurological deficits. This study aimed to investigate the association between comorbidities like diabetes mellitus (DM) and obesity and quality of life (QOL) in preoperative patients with DCM, and to examine the distribution of pain and numbness.

**Methods:**

A cross‐sectional study with 86 preoperative patients with DCM was conducted. Patient‐reported outcome measures (PROMs) including Core Outcome Measure Index for the neck (COMI‐Neck), Neck Disability Index (NDI), EQ‐5D‐3L, SF‐12v2 assessed QOL, and baseline characteristics were collected. Patients were categorized by diabetic and obesity status, resulting in 17 with and 69 without DM, and 27 obese, 59 nonobese patients. In the statistical analysis, we compared PROMs and baseline characteristics, and conducted MANCOVA to investigate the association of DM and obesity with PROMs.

**Results:**

The study found no significant differences in preoperative QOL between patients with and without DM or obesity. Additionally, the results of MANCOVA indicated that neither DM nor obesity alone, nor their combination, had an association with the total scores of PROMs. In each group, the Symptom‐specific well‐being score on the COMI‐Neck was notably high, implying distressing current symptoms (median: 10). On the NDI, the median score for pain intensity, lifting, work, and recreation subitems was 3. Pain was predominantly reported in the neck and lower back, while numbness was more prevalent in the peripheral regions of the upper and lower limbs.

**Conclusion:**

Preoperative QOL was not significantly affected by the presence of DM and/or obesity. DCM‐related symptoms may mask the associations with these comorbidities. Regardless of the preoperative condition, it is important to address the PROMs items that posed challenges before surgery.

## Key points

- Degenerative cervical myelopathy (DCM) significantly affects patients' quality of life.
- This study examines the association between diabetes mellitus and obesity with quality of life in preoperative patients with DCM, an underexplored area.
- Limited associations of diabetes mellitus and obesity with preoperative quality of life suggest prioritizing interventions targeting DCM‐related symptoms in preoperative counseling and postoperative care.

## 1 INTRODUCTION

Degenerative cervical myelopathy (DCM) is a debilitating condition characterized by compression of the cervical spinal cord, resulting in a range of neurological deficits.^1^, ^2^ Surgical intervention for DCM has demonstrable benefits with respect to symptom alleviation, functional recovery, and overall well‐being, underscoring its pivotal role in enhancing patients' quality of life (QOL).^3^, ^4^ However, the extent of these improvements hinges upon various preoperative factors, including individual characteristics and symptom severity.^3^, ^5^, ^6^

Among these important preoperative factors, the presence of comorbidities such as diabetes mellitus (DM) and obesity stands out as influential determinants of surgical outcomes.^7^, ^8^, ^9^ Individuals dealing with DM often face challenges that extend beyond their physical health, impacting on their QOL in multiple ways. Meta‐analyses have illustrated the association between a history of DM and suboptimal recovery following surgical intervention for DCM, revealing reduced changes in Japanese Orthopedic Association scores and an increased vulnerability to postoperative complications.^10^ Similarly, obesity has emerged as a significant player in the realm of preoperative considerations for patients with DCM.^11^ Notably, obese individuals with physical disabilities experience compromised health‐related QOL,^12^ further emphasizing the importance of exploring the intricate relationship between obesity and surgical outcomes. Within the context of DCM, obesity has been correlated with diminished postoperative improvements in both physical and mental health.^13^ Moreover, obesity amplifies the risk of postoperative complications, including infections, deep vein thrombosis, and pulmonary embolism.^14^ It has been shown that patients with DCM who also have DM or obesity are more likely to experience persistent neck pain postoperatively and have a higher incidence of high‐impact chronic pain even 3 months after surgery. These patients also tend to show less improvement in scores such as the Neck Disability Index (NDI) and EuroQOL‐5 dimension.^15^, ^16^ Despite reports indicating the association of these comorbidities with postoperative outcomes and QOL, there is limited literature regarding preoperative QOL. Understanding that the presence of comorbidities can increase difficulties in activities of daily living and contribute to anxiety about movements, it becomes evident that targeted interventions should be emphasized in preoperative counseling and postoperative rehabilitation.

On the other hand, factors such as pain and numbness may contribute to QOL in patients with DCM. It has been reported that patients with DCM prioritize pain recovery.^17^ Despite this, reports on pain in patients with DCM are often limited to pain and numbness in the neck and upper limbs, with few accounts of its association with widespread impact on the entire body.^18^ Traumatic spinal cord injury induces central sensitization, resulting in pain emerging in remote regions and causing complex systemic pain experiences in both humans and animal models.^19^ Although the pathophysiology between spinal cord injury and DCM is not identical,^20^, ^21^ there are overlapping aspects, suggesting the possibility that pain may be widespread throughout the body in patients with DCM.

This study aims to investigate the hypothesis that comorbidities like DM or obesity are associated with lower preoperative QOL in patients with DCM. Additionally, the study seeks to deepen understanding of the distribution of pain and numbness that could potentially affect QOL. The primary hypothesis was that individuals with DM and obesity would have a lower preoperative QOL compared to those without these comorbidities. Specifically, it was anticipated that the challenges posed by DCM‐related tetraplegia, coupled with the presence of DM and obesity, would synergistically contribute to a greater burden on patients' well‐being. By employing a comprehensive range of patient‐reported outcome measures (PROMs), including the Japanese Core Outcome Index for the Neck (COMI‐Neck), NDI, EuroQOL‐5 dimension‐3 level (EQ‐5D‐3L), and Short Form‐12 version 2 (SF‐12v2),^22^ the aim was to gain a nuanced understanding of various dimensions of patients' QOL before impending cervical spine surgery. As a second hypothesis, it was considered that compression of the spinal cord could lead to the spread of pain throughout the body.

## 2 METHODS

### 2.1 Study design and participants

This cross‐sectional study, approved by the Institutional Review Board of the Saitama Medical University Medical Center, Saitama Medical University (1969‐III), aimed to assess the QOL in preoperative patients with DCM, comparing those with and without DM and obesity, and adhered to the Strengthening the Reporting of Observational Studies in Epidemiology statement. Due to its retrospective nature, individual consent was not sought. However, a disclosure statement with contact information for data refusal was provided on the website, and data from patients who opted out were not included in the analysis. The study was conducted between April 2018 and June 2022, during which 107 consecutive patients scheduled for cervical spine surgery due to DCM were enrolled. Participants were chosen based on their ability to complete the necessary PROMs. Ultimately, 86 out of the initial 107 patients (80.3%) completed all PROMs and were included in the final analysis.

### 2.2 Data collection

**Figure 1:**
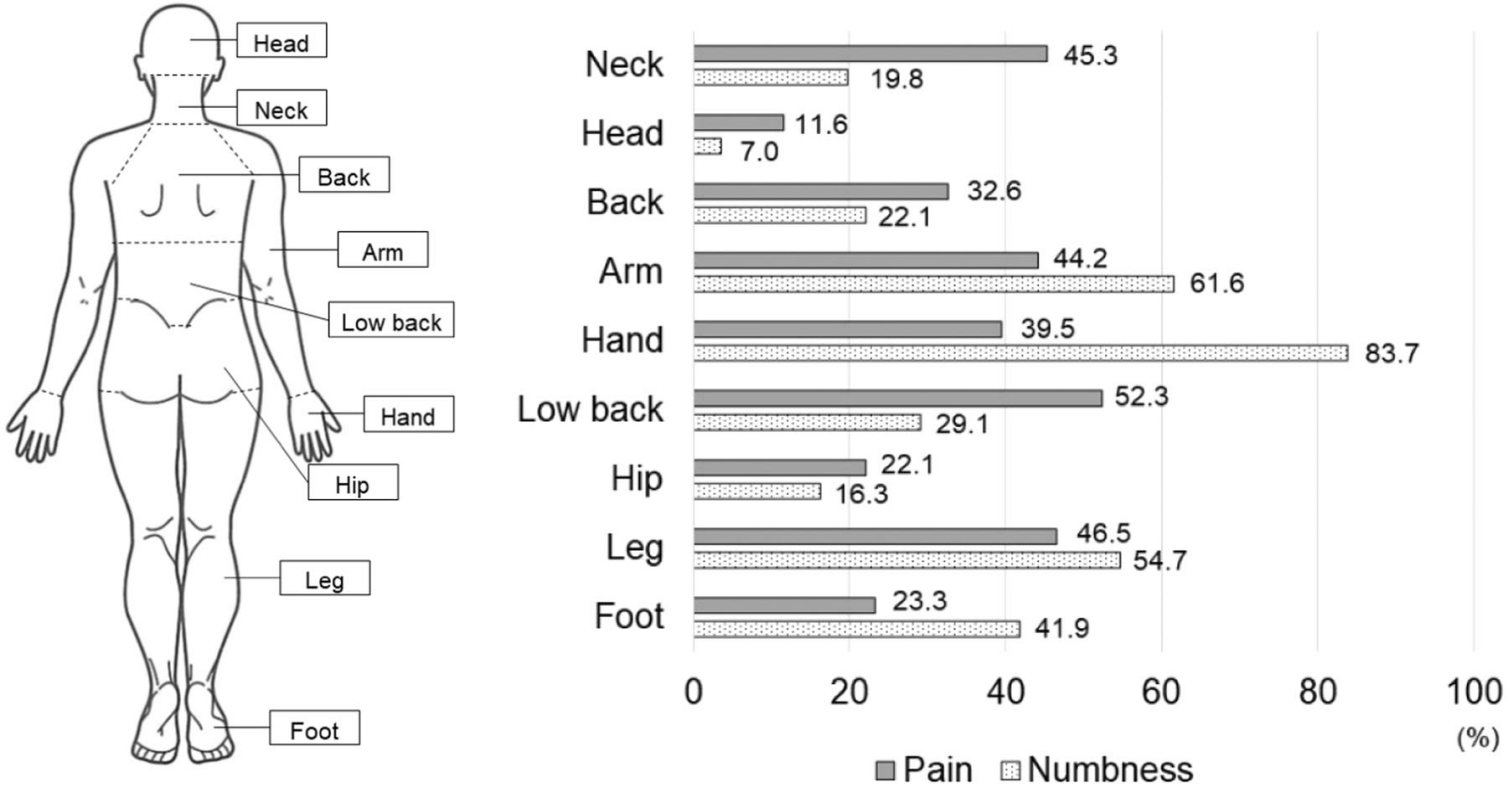
Distribution of pain and numbness. The figure illustrates the proportions of pain and numbness with the Numerical Rating Scale scores of 3 or higher.

The study gathered baseline demographic and clinical data from the participants, encompassing age, gender, body mass index (BMI: kg/m²), American Society of Anesthesiologists (ASA) classification, smoking habits, and medical history of DM, hypertension, hyperlipidemia, and arrhythmias. These variables, inclusive of comorbidities, were obtained from the medical records of each patient as recorded by the attending physician. Additionally, pain and numbness in the neck, head, back, arm, hand, low back, hip, leg, and foot were assessed using the Numerical Rating Scale (NRS) (Figure 1). The threshold for the presence of pain or numbness was set at a score of 3 or higher on the NRS.^23^

### 2.3 Patient‐reported outcome measures

To evaluate QOL among preoperative patients, we employed a series of PROMs that encompassed various dimensions of their well‐being. These assessments were administered before the scheduled cervical spine surgery. The Japanese COMI‐Neck questionnaire was utilized as a tool to gauge the severity of neck pain.^24^, ^25^ This comprehensive questionnaire encompasses multiple domains, including pain, functional limitations, symptom‐specific well‐being (SSWB), overall QOL, and disability. Each domain is rated on a scale from 0 to 10, and an aggregate score is calculated by averaging the individual domain scores. Higher scores on this scale indicate a more pronounced negative impact of spinal disease on the patient's life. For the assessment of neck disability's impact on daily activities, the Japanese modified NDI was employed.^26^ The index consists of seven items pertaining to daily functioning, two items related to pain, and one item focusing on concentration. Each item is scored on a scale ranging from 0 to 5, with the cumulative score indicating the level of impairment in daily activities. A higher score signifies greater limitations in daily activities. health‐related QOL, encompassing various aspects, was evaluated using the EQ‐5D‐3L.^27^, ^28^ This instrument assesses five dimensions: mobility, self‐care, usual activities, pain/discomfort, and anxiety/depression. Participants rate each dimension on a scale from 1 to 3, with higher scores indicating a higher level of problems related to that dimension. The five‐item scores contribute to the calculation of a utility score that reflects overall health‐related QOL. Furthermore, the SF‐12v2 questionnaire was employed to holistically measure the physical and mental well‐being of participants. This questionnaire encompasses eight health concepts, including Physical Functioning, Role Physical, Bodily Pain, General Health, Vitality, Social Functioning, Role Emotional, and Mental Health. Three summary scores are derived from these items: the Physical Component Score, the Mental Component Score, and the Role Component Score. These scores, when compared to national norm‐based scoring, provide insights into participants' overall health status.^29^, ^30^ Taking into account biases due to fatigue and stress, the order of evaluation of these PROMs was randomized. To more accurately capture patients' responses to these PROMs, patients completed them in a separate room away from the physician's presence. In cases of any questions or concerns, dedicated administrative staff provided assistance.

### 2.4 Statistical analysis

Descriptive statistics were used to summarize the baseline characteristics of the study population. To compare basic information and preoperative PROMs between groups, the Mann–Whitney *U* test and the *χ* ^2^ test were employed for continuous and categorical variables, respectively. All statistical analyses were performed using IBM SPSS Statistics for Windows, Version 29.0 (Armonk, NY: IBM Corp Released 2017), with a significance level set at *p* = 0.05. In the event of significant differences in PROMs between groups, a multiple regression analysis was employed using the forced entry method to evaluate the association between baseline demographic details and the outcomes. Furthermore, MANCOVA was conducted to examine the relationship between DM, obesity, and the total scores of the six PROMs (COMI‐Neck, NDI, EQ‐5D‐5L, and SF‐12v2 [PCS, MCS, and RCS]). Sample size determination was guided by a rule‐of‐thumb estimate, which recommends including at least 10–15 participants per covariate in the regression models.^31^ For the MANCOVA analysis, with six covariates, this guideline suggests a required sample size of 60–95 participants. Our study included a total of 86 participants, meeting the minimum sample size requirement to ensure sufficient statistical power. While this sample size is adequate, we recognize that a larger sample would enhance the power and reliability of our findings. Future studies should aim to include a larger sample to further validate these associations.

## 3 RESULTS

The patients were divided into groups based on the presence of DM or obesity (BMI > 25). Consequently 17 patients were in the DM group, 69 patients in the non‐DM group/27 patients in the obesity group, and 59 patients in the nonobesity group. The distribution of pain and numbness in all patients is illustrated in Figure 1. The highest proportions of individuals reporting pain were in the following order: Low back (52.3%), leg (46.5%), neck (45.3%), arm (44.2%), and hand (39.5%). The highest proportions of individuals reporting numbness were in the following order: Hand (83.7%), arm (61.6%), leg (54.7%), and foot (41.9%).

### 3.1 Comparison between groups by presence of diabetes and obesity

The results of the descriptive and inferential statistics are presented in Tables 1, 2, 3, 4. The DM group exhibited a higher mean age (95% confidence interval [CI]: DM; 69.2–78.0, non‐DM; 63.7–69.2) and a higher percentage of smokers compared to the non‐DM group. The PROMs indicated lower values for the NDI‐Sleeping item in the DM group (95% CI: DM; 0.11–0.94, non‐DM; 1.07–1.77). While the obesity group had a younger mean age than the nonobesity group (95% CI: obesity; 57.2–67.2, nonobesity; 68.0–73.0), no significant differences were observed in PROMs summary scores or individual subitems. In the nonobesity group, the severity of hand numbness was greater when compared to the obesity group (*p* = 0.025). However, with a power (1‐β) of 0.55, statistical significance was not achieved.

**Table 1:** Demographic and clinical characteristics for diabetes mellitus and nondiabetes mellitus groups.

	Diabetes mellitus (*n* = 17)	Nondiabetes mellitus (*n* = 69)	*p*
Age (years)	73.6 ± 8.6 (76.0)	66.5 ± 11.4 (68.0)	0.008
Sex, *n* (male/female [%])	13 (76.5)/4 (23.5)	52 (75.4)/17 (24.6)	0.60
BMI (kg/m^2^)	24.0 ± 3.2 (23.0)	23.7 ± 3.5 (23.1)	0.60
ASA classification (1/2/3 [%])	0 (0.0)/15 (88.2)/2 (11.8)	9 (13.0)/52 (75.4)/8 (11.6)	0.35
Smoke, *n* (%)	16 (94.1)/1 (5.9)	47 (68.1)/22 (31.9)	0.02
Hypertension, *n* (%)	10 (58.8)/7 (41.2)	27 (39.1)/42 (60.9)	0.14
Hyperlipidemia, *n* (%)	2 (11.8)/15 (88.2)	7 (10.1)/62 (89.9)	0.57
Arrythmia, *n* (%)	0 (0.0)/17 (100)	5 (7.2)/64 (92.8)	0.32
NRS (pain intensity)			
Neck	2.5 ± 2.5 (2)	2.6 ± 2.7 (2)	0.90
Head	0.6 ± 1.3 (0)	0.8 ± 1.8 (0)	0.77
Back	1.2 ± 2.2 (0)	2.1 ± 2.4 (1)	0.16
Arm	2.3 ± 3.0 (0)	2.5 ± 2.7 (1)	0.57
Hand	2.4 ± 3.2 (1)	2.6 ± 3.2 (1)	0.90
Low back	2.7 ± 3.0 (2)	3.2 ± 3.0 (3)	0.47
Hip	1.0 ± 2.2 (0)	1.4 ± 2.5 (0)	0.43
Leg	3.1 ± 3.2 (3)	2.7 ± 3.1 (2)	0.68
Foot	1.2 ± 2.1 (0)	1.3 ± 2.6 (0)	0.97
NRS (Numbness intensity)			
Neck	1.8 ± 2.4 (1)	1.1 ± 2.0 (0)	0.22
Head	0.1 ± 0.3 (0)	0.4 ± 1.2 (0)	0.44
Back	0.9 ± 1.7 (0)	1.3 ± 2.0 (0)	0.46
Arm	4.1 ± 2.8 (4)	3.8 ± 3.0 (4)	0.73
Hand	6.8 ± 2.4 (8)	5.7 ± 3.1 (7)	0.20
Low back	1.4 ± 2.2 (0)	1.9 ± 2.7 (0)	0.70
Hip	0.5 ± 1.4 (0)	1.3 ± 2.2 (0)	0.13
Leg	3.5 ± 3.4 (3)	3.6 ± 3.2 (3)	0.80
Foot	2.9 ± 3.1 (2)	2.9 ± 3.5 (1)	0.88

*Note*: Mean ± SD (median); Mann–Whitney *U* test and the *χ* ^2^ test. ASA, American Society of Anesthesiologists; BMI, body mass index; NRS, Numerical Rating Scale; PROMs, patients‐reported outcome measures.

**Table 2:** Comparison of PROMs items for diabetes mellitus and nondiabetes mellitus groups.

	Diabetes mellitus (*n* = 17)	Nondiabetes mellitus (*n* = 69)	*p*
COMI‐Neck			
Summary score	6.5 ± 2.2 (6.9)	6.3 ± 2.0 (6.7)	0.69
Pain	4.0 ± 3.5 (5.0)	4.3 ± 2.9 (4.0)	0.66
Function	6.3 ± 3.6 (7.5)	5.5 ± 3.5 (7.5)	0.36
SSWB	8.4 ± 3.1 (10.0)	9.2 ± 2.0 (10.0)	0.29
General QOL	7.1 ± 2.0 (7.5)	7.4 ± 2.1 (7.5)	0.50
Disability	6.7 ± 3.4 (6.25)	5.3 ± 3.9 (5.0)	0.16
NDI			
NDI‐score	41.3 ± 11.7 (42)	43.1 ± 17.8 (44)	0.71
Pain intensity	2.5 ± 0.9 (3)	2.8 ± 1.1 (3)	0.31
Personal care	2.5 ± 1.2 (2)	2.3 ± 1.1 (2)	0.44
Lifting	3.2 ± 1.3 (3)	2.9 ± 1.3 (3)	0.40
Reading	2.1 ± 1.3 (3)	2.2 ± 1.4 (3)	0.65
Headaches	0.4 ± 0.7 (0)	0.5 ± 1.0 (0)	0.79
Concentration	1.9 ± 1.1 (2)	2.0 ± 1.3 (2)	0.92
Work	2.5 ± 1.7 (2)	2.3 ± 1.5 (2)	0.56
Driving	1.6 ± 1.5 (1)	2.2 ± 1.5 (2)	0.19
Sleeping	0.5 ± 0.8 (0)	1.4 ± 1.4 (1)	0.02
Recreation	3.4 ± 1.6 (4)	3.1 ± 1.6 (3)	0.51
EQ‐5D			
Utility score	0.5 ± 0.2 (0.5)	0.5 ± 0.2 (0.5)	0.97
Mobility	2.0 ± 0.6 (2.0)	1.9 ± 0.5 (2.0)	0.68
Self‐care	2.0 ± 0.6 (2.0)	1.8 ± 0.6 (2.0)	0.28
Usual activities	2.3 ± 0.6 (2.0)	2.1 ± 0.5 (2.0)	0.26
Pain/discomfort	2.2 ± 0.5 (2.0)	2.2 ± 0.6 (2.0)	0.81
Anxiety/depression	1.7 ± 0.6 (2.0)	1.8 ± 0.6 (2.0)	0.33
SF‐12v2			
PCS	20.7 ± 12.6 (17.7)	22.8 ± 13.5 (21.3)	0.62
MCS	47.8 ± 10.9 (44.0)	50.3 ± 11.4 (48.8)	0.30
RCS	32.1 ± 13.5 (30.5)	34.5 ± 14.7 (34.9)	0.59
Physical functioning	14.4 ± 14.7 (2.7)	22.7 ± 16.5 (16.0)	0.055
Role physical	19.7 ± 11.9 (17.5)	20.3 ± 13.7 (17.5)	0.97
Bodily pain	30.5 ± 13.7 (23.9)	29.1 ± 13.3 (23.9)	0.72
General health	36.1 ± 10.4 (35.7)	38.2 ± 11.2 (35.7)	0.51
Vitality	37.7 ± 8.8 (38.5)	41.1 ± 10.9 (38.5)	0.32
Social function	31.0 ± 16.0 (33.7)	37.3 ± 15.3 (33.7)	0.13
Role emotional	31.6 ± 16.1 (32.0)	32.7 ± 15.4 (32.0)	0.77
Mental health	36.2 ± 13.5 (33.8)	41.0 ± 13.5 (39.8)	0.17

*Note*: Mean ± SD (median); Mann–Whitney *U* test; Abbreviations: COMI‐Neck, Core Outcome Measure Index for neck; EQ‐5D, EuroQol‐5 dimension; MCS, mental component summary; NDI, Neck Disability Index; PCS, physical component summary; PROMs, patients‐reported outcome measures; QOL, quality of life; RCS, role component summary; SF‐12v2, short form‐12; SSWB, symptom‐specific well‐being.

**Table 3:** Demographic and clinical characteristics for each of the obesity and nonobesity groups.

	Obesity (*n* = 27)	Nonobesity (*n* = 59)	*p*
Age (years)	62.2 ± 12.5 (64)	70.5 ± 9.6 (72)	0.003
Sex, *n* (male/female [%])	22 (81.5)/5 (18.5)	43 (72.9)/16 (27.1)	0.39
BMI (kg/m^2^)	27.8 ± 2.6 (27.0)	22.0 ± 1.7 (22.0)	‐
ASA classification (1/2/3 [%])	3 (11.1)/21 (77.8)/3 (11.1)	6 (10.2)/46 (78.0)/7 (11.9)	1.00
Smoke, *n* (%)	20 (74.1)/7 (25.9)	43 (72.9)/16 (27.1)	0.91
Diabetes mellitus, *n* (%)	4 (14.8)/23 (85.2)	13 (22.0)/46 (78.0)	0.44
Hypertension, *n* (%)	12 (44.4)/15 (55.6)	25 (42.4)/34 (57.6)	0.86
Hyperlipidemia, *n* (%)	2 (7.4)/25 (92.6)	7 (11.9)/52 (88.1)	0.42
Arrythmia, *n* (%)	1 (3.7)/26 (96.3)	4 (6.8)/55 (93.2)	0.50
NRS (Pain intensity)			
Neck	2.1 ± 2.2 (2)	2.8 ± 2.8 (3)	0.28
Head	0.5 ± 1.2 (0)	0.9 ± 1.9 (0)	0.54
Back	1.7 ± 2.2 (1)	2.0 ± 2.5 (1)	0.67
Arm	2.6 ± 2.5 (3)	2.4 ± 2.9 (1)	0.49
Hand	2.2 ± 3.0 (1)	2.7 ± 3.3 (1)	0.57
Low back	3.2 ± 3.3 (2)	3.0 ± 2.8 (3)	0.82
Hip	1.4 ± 2.5 (0)	1.3 ± 2.4 (0)	0.92
Leg	2.6 ± 3.3 (1)	2.9 ± 3.0 (2)	0.55
Foot	0.9 ± 2.3 (0)	1.5 ± 2.6 (0)	0.25
NRS (Numbness intensity)			
Neck	0.9 ± 1.8 (0)	1.4 ± 2.2 (0)	0.32
Head	0.2 ± 0.7 (0)	0.4 ± 1.2 (0)	0.34
Back	1.4 ± 2.3 (0)	1.1 ± 1.7 (0)	0.83
Arm	3.3 ± 2.6 (3)	4.1 ± 3.0 (4)	0.29
Hand	4.9 ± 3.1 (4)	6.4 ± 2.9 (7)	0.03*
Low back	1.8 ± 3.0 (0)	1.7 ± 2.5 (0)	0.91
Hip	1.0 ± 2.4 (0)	1.2 ± 2.0 (0)	0.18
Leg	3.0 ± 3.2 (2)	3.9 ± 3.3 (4)	0.29
Foot	2.7 ± 3.4 (1)	3.0 ± 3.4 (1)	0.69

*Note*: Mean ± SD (median); Mann–Whitney *U* test and the *χ* ^2^ test; Abbreviations: ASA, American Society of Anesthesiologists; BMI, body mass index; NRS, Numerical Rating Scale; PROMs, patients‐reported outcome measures.

* *p* < 0.05.

**Table 4:** Comparison of PROMs items for the obesity and nonobesity groups.

	Obesity (*n* = 27)	Nonobesity (*n* = 59)	*p*
COMI‐Neck			
Summary score	6.5 ± 2.2 (6.9)	6.3 ± 2.0 (6.7)	0.69
Pain	4.0 ± 3.5 (5.0)	4.3 ± 2.9 (4.0)	0.66
Function	6.3 ± 3.6 (7.5)	5.5 ± 3.5 (7.5)	0.36
SSWB	8.4 ± 3.1 (10.0)	9.2 ± 2.0 (10.0)	0.29
General QOL	7.1 ± 2.0 (7.5)	7.4 ± 2.1 (7.5)	0.50
Disability	6.7 ± 3.4 (6.25)	5.3 ± 3.9 (5.0)	0.16
NDI			
NDI‐score	41.3 ± 11.7 (42)	43.1 ± 17.8 (44)	0.71
Pain intensity	2.5 ± 0.9 (3)	2.8 ± 1.1 (3)	0.31
Personal care	2.5 ± 1.2 (2)	2.3 ± 1.1 (2)	0.44
Lifting	3.2 ± 1.3 (3)	2.9 ± 1.3 (3)	0.40
Reading	2.1 ± 1.3 (3)	2.2 ± 1.4 (3)	0.65
Headaches	0.4 ± 0.7 (0)	0.5 ± 1.0 (0)	0.79
Concentration	1.9 ± 1.1 (2)	2.0 ± 1.3 (2)	0.92
Work	2.5 ± 1.7 (2)	2.3 ± 1.5 (2)	0.56
Driving	1.6 ± 1.5 (1)	2.2 ± 1.5 (2)	0.19
Sleeping	0.5 ± 0.8 (0)	1.4 ± 1.4 (1)	0.02
Recreation	3.4 ± 1.6 (4)	3.1 ± 1.6 (3)	0.51
EQ‐5D			
Utility score	0.5 ± 0.2 (0.5)	0.5 ± 0.2 (0.5)	0.91
Mobility	1.9 ± 0.5 (2.0)	2.0 ± 0.5 (2.0)	0.72
Self‐care	1.8 ± 0.6 (2.0)	1.9 ± 0.6 (2.0)	0.59
Usual activities	2.2 ± 0.5 (2.0)	2.2 ± 0.6 (2.0)	0.85
Pain/discomfort	2.2 ± 0.6 (2.0)	2.2 ± 0.6 (2.0)	0.75
Anxiety/depression	1.9 ± 0.7 (2.0)	1.7 ± 0.6 (2.0)	0.32
SF‐12v2			
PCS	26.1 ± 13.9 (29.9)	20.7 ± 12.8 (19.0)	0.09
MCS	50.0 ± 12.6 (47.2)	49.7 ± 10.7 (48.9)	0.89
RCS	32.6 ± 15.8 (32.3)	34.7 ± 13.8 (34.6)	0.71
Physical functioning	25.3 ± 15.9 (29.2)	19.1 ± 16.4 (16.0)	0.07
Role physical	20.1 ± 12.8 (17.5)	20.3 ± 13.7 (17.5)	0.93
Bodily pain	28.9 ± 12.5 (23.9)	30.0 ± 13.8 (23.9)	0.92
General health	41.2 ± 12.7 (35.7)	36.2 ± 9.9 (35.7)	0.09
Vitality	41.2 ± 12.0 (38.5)	40.2 ± 9.9 (38.5)	0.85
Social function	37.9 ± 16.9 (33.7)	35.2 ± 15.0 (33.7)	0.39
Role emotional	33.2 ± 14.8 (32.0)	32.6 ± 15.9 (32.0)	0.93
Mental health	38.9 ± 14.9 (39.8)	41.0 ± 13.0 (39.8)	0.63

*Note*: Mean ± SD (median); Mann–Whitney *U* test; Abbreviations: COMI‐Neck, Core Outcome Measure Index for neck; EQ‐5D, EuroQol‐5 dimension; MCS, mental component summary; NDI, Neck Disability Index; PCS, physical component summary; PROMs, patients‐reported outcome measures; QOL, quality of life; RCS, role component summary; SF‐12v2, short form‐12; SSWB, symptom‐specific well‐being.

Irrespective of DM or obesity status, in the Japanese COMI‐Neck, mean scores for Function, SSWB, General QOL, and Disability were above 5. Notably, the median score for SSWB was 10, indicating lower well‐being. Within the NDI, subitems such as Pain intensity, Lifting, Work, and Recreation had a median value of 3, while other sub‐items scored 2 or lower. Regarding the EQ‐5D‐3L, all sub‐items had a median value of 2 (indicating some problems). The SF‐12v2 scores exhibited generally low values, particularly for Physical Functioning, Role Physical, and Bodily Pain, all of which had median scores below 30.

### 3.2 Multiple regression analysis and MANCOVA

For NDI‐Sleeping, which showed significant differences between those with and without DM, multiple regression analysis was performed using the forced entry method with age, DM status, and smoking status as independent variables. For the NDI‐Sleeping item, ANOVA was significant at *p* = 0.026, indicating a statistically significant model. However, age (standardized coefficient *β* = −0.088, *p* = 0.439), DM status (standardized coefficient *β* = 0.199, *p* = 0.073), and smoke status (standardized coefficient *β* = 0.161, *p* = 0.158) had no significant effect on the scores of NDI‐Sleeping items. These statistical analyses showed that preoperative QOL was not affected by DM. In MANCOVA, there was no significant effect of either DM or obesity alone, or the combination of DM and obesity, on the total scores of the four PROMs (Table 5).

**Table 5:** Effect of diabetes mellitus and obesity on patients‐reported outcome measures and subscale using MANCOVA.

Condition	Dependent variables	*F*‐value	*p*	Partial *η* ^2^
Diabetes mellitus	COMI‐Neck core index	0.39	0.535	0.005
[df: 1, 86]	NDI score	<0.01	0.989	<0.001
	EQ‐5D‐3L	0.72	0.399	0.009
	SF‐12v2: PCS	<0.01	0.986	<0.001
	SF‐12v2: MCS	0.79	0.378	0.009
	SF‐12v2: RCS	0.85	0.360	0.010
Obesity	COMI‐Neck core index	0.03	0.957	<0.001
[df: 1, 86]	ODI score	0.04	0.853	<0.001
	EQ‐5D‐3L	0.07	0.797	0.001
	SF‐12v2: PCS	3.40	0.069	0.040
	SF‐12v2: MCS	0.05	0.829	0.001
	SF‐12v2: RCS	0.88	0.351	0.011
DM and obesity	COMI‐Neck core index	0.77	0.383	0.009
[df: 1, 86]	ODI score	0.53	0.470	0.006
	EQ‐5D‐3L	0.10	0.748	0.001
	SF‐12v2: PCS	0.70	0.404	0.009
	SF‐12v2: MCS	0.15	0.702	0.002
	SF‐12v2: RCS	0.46	0.501	0.006

*Note*: The MANCOVA results indicate a significant overall effect for PROMs (DM: Wilk's Λ = 0.947, partial *η* ^2^ = 0.053, *p* = 0.640, obesity: Wilk's Λ = 0.942, partial *η* ^2^ = 0.058, *p* = 0.579, DM and obesity: Wilk's Λ = 0.972, partial *η* ^2^ = 0.028, *p* = 0.894).

## 4 DISCUSSION

The aim of this study was to investigate the potential association between comorbidities like DM and obesity and preoperative QOL in patients diagnosed with DCM and awaiting surgery, as well as to examine the distribution of pain and numbness. While DM and obesity have been associated with postoperative complications and functional outcomes, we sought to explore their association with patients' preoperative well‐being. Contrary to our initial hypothesis, the findings of this study reveal that these comorbidities did not significantly affect preoperative QOL. On the other hand, as hypothesized, pain and numbness were distributed throughout the body. The highest frequency of pain reports was in the low back and neck, while numbness was more commonly reported in the hand and arm.

The results of multiple regression analysis and MANCOVA indicated that there was no significant association between DM and obesity and preoperative QOL. This suggests that DCM‐related symptoms may have had a dominant association with the responses in preoperative PROMs. The debilitating consequences of spinal cord compression in DCM, including pain, motor dysfunction, and limitations in daily activities,^32^ likely overshadowed the potential effects of comorbidities on preoperative QOL assessments. In previous reports, 50%–66% of patients with DM were documented to develop Peripheral neuropathy at some point in their lifetime, leading to numbness in the extremities.^33^, ^34^ While it remains unclear whether the DM group in our study had concurrent peripheral neuropathy associated with DM, the comparable ratings of numbness and pain assessed by the NRS suggest that tingling sensations and pain in the extremities, possibly arising from DCM, might have been more pronounced. Considering the DM group in our study had a higher proportion of known risk factors for peripheral neuropathy, such as older age and a prevalence of smoking habits, it is crucial to continue monitoring symptoms related to DM even after alleviating DCM‐related symptoms through surgery. This underscores the importance of ongoing vigilance and management of DM‐related symptoms postoperatively, recognizing the potential persistence of diabetic neuropathy factors. On the other hand, obesity has been shown to be associated not only with DM but also with low back pain.^35^, ^36^ The prevalence and intensity of low back pain are reported to be higher in individuals with obesity.^37^ However, in the obesity group of this study, these factors were not found to be correlated. While it is possible for patients with DCM to experience coexisting low back pain,^38^ this study revealed that the nonobesity group had low back pain of comparable intensity to neck pain. This finding may have obscured the association with obesity. Therefore, healthcare professionals should not only focus on DCM‐specific symptoms but also consider symptoms of low back pain. However, this study did not investigate the history of conditions such as lumbar disc herniation or lumbar spinal stenosis, nor did it conduct physical function tests to assess the cause of lower back pain. Future research should include an evaluation of the lumbar region in patients with DCM who experience lower back pain. Additionally, the unique context of the preoperative phase might also contribute to these findings. Patients facing imminent surgical intervention for DCM might magnify symptoms directly related to their spinal condition, potentially downplaying the association with other comorbidities. Moreover, the anticipation of surgery, with its associated uncertainties and anxieties, might further diminish the association between DM, obesity, and preoperative QOL.^39^ While our study's results contradict previous literature that underscores the negative effects of DM and obesity on postoperative functional recovery and complications,^7^, ^8^, ^9^ they support the notion that the preoperative setting has the potential to influence patients' perceptions. These findings emphasize the complex interplay of various factors in shaping patients' experiences and underscore the significance of taking into account the specific clinical context when interpreting QOL assessments.

In our sample, numbness was concentrated in the peripheral regions of the limbs, while pain was reported throughout the body, not limited to the neck and upper extremities. Kutch et al. have reported that the extensive anatomical distribution of pain is associated with severity, particularly in conditions like fibromyalgia and pelvic pain, linked to central sensitization of pain.^40^ Additionally, in traumatic spinal cord injury, the anatomical distribution and severity of pain have been shown to be associated with impairment of descending pain inhibition and spinal cord hyperexcitability in pain processing.^41^ While the relationship between pain and numbness in specific body regions and QOL remains unclear, it is evident that certain individuals undergo distress beyond that identified by conventional assessments. These factors may contribute to a significant decline in preoperative QOL for patients with DCM.^42^ Given that the foremost objective of surgery for patients is pain improvement,^17^ it is important to consider this preoperative condition in the postoperative care and rehabilitation of patients with DCM.

This study has several limitations that should be acknowledged. Firstly, due to its retrospective nature, we were unable to capture the full spectrum of severity for each comorbidity, particularly DM. The severity of DM can vary widely among patients, and this variability was not accounted for in our analysis. We also did not include detailed blood data, such as HbA1c levels or the duration of DM, which could provide additional insights into the relationship between DM severity and preoperative QOL. Additionally, we did not explore characteristics specific to obese patients with a higher BMI, which could affect the interpretation of our findings regarding obesity. The outcome measures we selected may have precluded the detection of more subtle effects of comorbidities on preoperative QOL. The retrospective design of the study limits the ability to establish causality. The sample size, while adequate for detecting some differences, may still be insufficient for more nuanced subgroup analyses. Furthermore, the unique context of the preoperative phase might affect patients' perceptions of their QOL, potentially overshadowing the associations with comorbidities. As patients anticipate surgery, their focus may be more on the impending procedure and less on other health conditions, which could affect their responses to the PROMs. Additionally, one significant limitation of our study is the inherent selection bias due to the inclusion of only preoperative patients scheduled for surgery, all of whom had a certain level of disability or impairment. This homogeneity likely limited our ability to detect significant associations between comorbidities such as DM and obesity and preoperative QOL. Future studies should consider including a broader spectrum of patients with DCM, including nonsurgical candidates, to better understand the impact of these comorbidities across different levels of disability. Moreover, attributing pain distribution solely to DCM in older adults with multiple comorbidities is challenging, as these patients often have overlapping musculoskeletal disorders. Previous studies have reported that up to 58% of patients with DCM may also have tandem stenosis, which is associated with poorer postoperative outcomes.^43^ However, our study did not investigate the condition of the lumbar spine, making it unclear whether these patients also had tandem stenosis. It has also been reported that even DCM alone can result in widespread pain.^18^ Therefore, as shown in our study, patients presenting with preoperative pain and numbness in the lower back and lower extremities should be monitored more closely postoperatively. Future research should investigate the distribution of pain and numbness, taking into account lumbar spine conditions. Furthermore, our study did not include important covariates such as the duration of symptoms or time since diagnosis, which can significantly affect patients' QOL. The lack of clinical indicators, such as physical exam findings, radiological measures (e.g., spinal canal diameter), and the number of spinal levels affected, further limits the comprehensiveness of our analysis. Finally, our study did not collect socioeconomic indicators that can influence healthcare access and decision‐making. Future research should include these variables to provide a more nuanced understanding of the factors affecting preoperative QOL in patients with DCM.

## 5 CONCLUSION

In conclusion, this study found that comorbidities like DM and obesity were not significantly associated with preoperative QOL in patients awaiting surgery for DCM. Despite the anticipated associations, DCM‐related symptoms appeared to outweigh the associations with comorbidities on preoperative QOL assessments. Pain and numbness were observed to be distributed throughout the body, emphasizing the complexity of patients' pain beyond conventional assessments. While the study has limitations, including sample size and design, it highlights the need for targeted interventions in preoperative counseling and postoperative care, acknowledging the specific clinical context when interpreting QOL assessments.

## AUTHOR CONTRIBUTIONS

**Yasuaki Mizoguchi**: Conceptualization; data curation; formal analysis; methodology; visualization; writing—original draft. **Kiyokazu Akasaka**: Conceptualization; data curation; methodology; supervision; writing—review and editing. **Kenta Suzuki**: Conceptualization; methodology; validation; writing—review and editing. **Fumihiko Kimura**: Methodology; validation; writing—review and editing. toby hall: validation; writing—review and editing. **Satoshi Ogihara**: Data curation; investigation; methodology; project administration; supervision; writing—review and editing.

## CONFLICT OF INTEREST STATEMENT

The authors declare no conflict of interest.

## ETHICS STATEMENT

All procedures performed in studies involving human participants were in accordance with the ethical standards of the institutional review board of the Saitama Medical University Medical Center, Saitama Medical University (1969‐III) and with the 1964 Helsinki Declaration and its later amendments or comparable ethical standards. Due to its retrospective nature, individual consent was not sought. However, a disclosure statement with contact information for data refusal was provided on the website, and data from patients who opted out were not included in the analysis.

## TRANSPARENCY STATEMENT

The lead author Kiyokazu Akasaka affirms that this manuscript is an honest, accurate, and transparent account of the study being reported; that no important aspects of the study have been omitted; and that any discrepancies from the study as planned (and, if relevant, registered) have been explained.

## Data Availability

The data that support the findings of this study are available from the corresponding author upon reasonable request.
